# Anticipated Barriers and Facilitators to the Implementation of an AI Tool Advising on the Placement of Mitigation Strategies to Improve Air Quality in the Hospital Setting: Qualitative Study

**DOI:** 10.2196/93420

**Published:** 2026-10-13

**Authors:** Yolanda Barrado-Martín, Sanjoli Mathur, Zarnie Khadjesari, Sigrún Eyrúnardóttir Clark, Cecilia Vindrola-Padros, Laurence B Lovat, Fiona Stevenson

**Affiliations:** 1 Research Department of Primary Care & Population Health, University College London, Upper 3rd Floor, Royal Free Campus, Rowland Hill St., London, England, NW3 2PF, United Kingdom, 44 2031088282; 2 School of Health Sciences, University of East Anglia, Norwich, England, United Kingdom; 3 Rapid Research Evaluation and Appraisal Lab (RREAL), Department of Targeted Intervention, University College London, London, England, United Kingdom; 4 Intelligent Systems in Gastroenterology & Healthcare Technology Lab (InSIGHT AI), Division of Surgery and Interventional Science, University College London, London, England, United Kingdom

**Keywords:** barriers and facilitators, artificial intelligence, implementation science, hospital, cross-infections, air filters, infection control, qualitative research, normalization process theory

## Abstract

**Background:**

AI is increasingly prevalent within health care; however, its implementation within the hospital setting may come with its own limitations, given the high-pressure and sensitive environment. Barriers and facilitators to the implementation of AI within hospitals have been largely explored quantitatively, and there is a scarcity of qualitative research on the implementation of AI tools to improve air quality.

**Objective:**

This study aims to qualitatively explore, through a normalization process theory (NPT) lens, the anticipated barriers and facilitators to the successful implementation of an AI tool to guide the placement of mitigation strategies to reduce airborne transmission of infections within the hospital setting. Specifically, we sought to (1) understand the day-to-day work of key hospital staff involved in reducing airborne transmission of infections, (2) identify potential barriers to the implementation of an AI tool to guide the placement of mitigation strategies in the hospital setting, and (3) recognize necessary conditions for the successful implementation of the AI tool within the hospital setting.

**Methods:**

We conducted ethnographic observations (n=4) and semistructured interviews (n=18) with hospital staff following preliminary one-on-one meetings and group discussions involving patients, members of the public, national leaders in ventilation working within academic and industry environments, and hospital staff (n=70). Data were collected between October 2025 and May 2026 in the United Kingdom. Interview transcripts, field notes, and summaries from preliminary wider engagement work activities were analyzed in NVivo (Lumivero; version 12) following codebook thematic analysis to facilitate confirmatory triangulation. Findings were organized according to the core constructs of NPT.

**Results:**

Hospital staff understanding of the AI tool and their preparedness to invest time in its implementation varied across those consulted. The anticipated work required by hospital staff to successfully implement the AI tool and its recommended mitigation strategies was identified as a significant challenge for its scalability. Differences in the ways of working among trusts and the competition for limited resources (collective action), and issues associated with hospitals’ physical estate (coherence) were seen as particularly problematic. The need for local champions (cognitive participation) was raised as a facilitator, as well as expert validation and the assessment of the AI tool’s effectiveness (reflexive monitoring).

**Conclusions:**

This preimplementation study suggests that the successful implementation of an AI tool to improve air quality in the hospital setting depends not only on the effectiveness demonstrated by the AI tool but also on the way trial outcomes are conveyed to hospital staff. Hospital staff anticipate an initial resistance to the AI tool’s implementation. Further, the impact of the AI tool and suggested mitigation strategies in workflows, costs, and staff and patient outcomes may be decisive.

## Introduction

AI is increasing its prevalence within health care and its adoption is supported by UK health policy, such as the National Health Service (NHS) 10-year plan and the creation of AI working groups to steer the use of AI and speed up its use within the NHS [1,2]. While AI’s predominant use has been in medical imaging [3], it has the potential to contribute to other areas, facilitating clinical decision-making, early diagnosis [4], and hospital documentation [5]. This aligns with the NHS ambition to shift “from analogue to digital,” prioritizing digital tools, including AI-driven solutions, to promote continuous monitoring and the shift “from sickness to prevention.” These shifts are intended to improve population health and the sustainability of health care [2].

AI-enabled tools have already shown potential in predicting hospital-acquired infections with high accuracy [6]. Early detection and intervention of airborne disease transmission can protect frontline workers and facilitate hospitals working at full capacity by reducing staff sickness. Our wider research team is developing and implementing an AI tool—the Artificial Intelligence Air Safety Tool (AISaT; University College London). AISaT will process data about the characteristics of a hospital room, such as its dimensions and the position of people in the room, to then produce recommendations on where best to position mitigation strategies within that room to reduce airborne infection transmission. Examples of mitigation strategies could include devices such as high-efficiency particulate air (HEPA) filters [7], or equipment such as screens, curtains [8], windows, or doors.

Despite the promise of AI in infection prevention and the potential of qualitative research to help facilitate implementation [9], few studies have focused on qualitatively exploring barriers and facilitators to implementation of AI within hospital settings [10,11]. This is known to be challenging [12], and identified barriers include the lack of trust, issues with generalizability, privacy and maintenance plans, or the previous lack of AI implementation guidelines [13], which have only been recently addressed [14]. When qualitative methods have been used, studies have mostly focused on AI looking to improve patient care directly (eg, by speeding diagnosis) [11,15].

The aim of this study was to explore, through a normalization process theory (NPT) lens [16,17], the anticipated barriers and facilitators to the successful implementation of AISaT, a tool that seeks to protect both staff and patients. This theory was chosen to map the anticipated requirements for the integration of AISaT into daily practice and facilitate trial delivery. By focusing on stakeholder experiences, including those of clinical, technical, and leadership teams, this work seeks to inform strategies that align with the NHS’s preventative care vision and support the normalization of such innovations in routine hospital practice.

The study sought to answer the question: how do relevant stakeholders perceive the implementation of an AI tool to guide the placement of mitigation strategies to reduce airborne transmission of infections within the hospital setting? Our objectives were to (1) understand the day-to-day work of key hospital staff involved in reducing airborne transmission of infections, (2) identify potential barriers to the implementation of an AI tool to guide the placement of mitigation strategies in the hospital setting, and (3) recognize necessary conditions for the successful implementation of the AI tool.

## Methods

### Design

Wider stakeholder engagement activities, drawing on findings from 2 systematic reviews in this area [18,19], were conducted. These comprised (1) an in-person patient advisory group (PAG) meeting, (2) an online workshop with national leaders, (3) follow-up conversations with national leaders, and (4) hospital staff based at 2 hospitals who would be involved in the subsequent clinical trial (trial registration number: ISRCTN12421486). Summaries containing data gathered from the informal conversations (wider stakeholder work) informed recruitment and topic guide development for formal data collection, while contributing to the joint analysis.

Drawing on insights from this wider stakeholder engagement, we conducted formal data collection (ethnographic observations and semistructured interviews) between October 15, 2025, and May 7, 2026, at a large teaching hospital in Greater London and a smaller teaching hospital in an urban town. The focus of this research was on the roles and responsibilities of hospital staff working to keep hospitals free from airborne disease transmission. Data collected at this stage were triangulated with aggregated summaries from the wider stakeholder engagement activities (informal conversations with hospital staff, national leaders in ventilation, and members of the public) as reflected in Figure 1.

**Figure 1.**
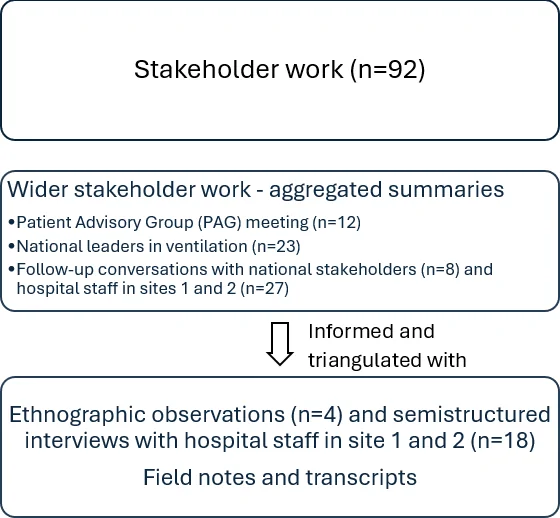
Summary of types of data collected.

Wider stakeholder engagement, and particularly informal interviews, compensated for relatively less formal semistructured interviews. Ethnographic observations complemented what was being said during semistructured interviews by allowing us to understand staff’s day-to-day work in ventilation. Formal semistructured interviews included questions raised in informal conversations and expanded on current practices, acceptability, and feasibility aspects. Data obtained formally and informally aligned well.

All data were collected before trialing of the AISaT system, so both informal discussions and formal data collection were based on a hypothetical future implementation of AISaT rather than on its actual use or implementation. The COREQ (Consolidated Criteria for Reporting Qualitative Research) checklist was used to support the presentation of methods and results [20] (Checklist 1).

For context, AISaT will require the user to introduce details on room geometry, airflow (including the nature of air conditioning if present), occupancy, and mitigations used (air filter, screens, fans, and so on). AISaT then uses computational fluid dynamics (CFD)–based simulations to generate 2D and 3D representations of the entire room, with a color chart showing ranked recommendations (from highest to lowest) based on the likelihood of chosen mitigations to reduce the number of respiratory particles. AISaT may only need to be used once in rooms where occupancy remains similar over time (eg, consulting rooms), while in areas where occupancy changes, it may need to be rerun accordingly. The ultimate decision to place the mitigation strategies where AISaT recommends lies with the human operating the device, who will be provided with alternatives and will need to avoid hazardous placements.

### Wider Stakeholder Engagement

The PAG was involved throughout the study. The PAG was consulted on the relevance and priority of clean air within hospitals, and their initial reactions to HEPA filters and to AISaT. Members of the group were recruited through advertisement on the National Institute for Health and Care Research (NIHR) People in Research website, charity connections, and established patient networks. The interaction with patients was led by SM, guided by Simon Watt and FS.

National leaders were invited to the online workshop and follow-up conversations were drawn from academic, industry, and health care sectors, through connections with members of the trial team and through online searches, because of their contributions to the field of ventilation. The workshop shared the project aims, sought first reactions about the trial, and helped to identify further national leaders in the field and key hospital job roles to target in ethnographic observations and interviews. It lasted 60 minutes to maximize participation by people working in the health service in high-pressure roles. The online workshop was led by YB-M and SM, with support from FS, ZK, and SEC.

The online workshop was supplemented by follow-up conversations, which allowed stakeholders to share more detail in relation to their views. Follow-up conversations were also conducted with people who could not join the workshop, and those who were identified through snowball sampling (n=8 [YB-M: n= 7; SM: n= 1]). After collecting information during the workshop about relevant hospital roles to engage with, the research team scheduled follow-up conversations with professionals in these roles (n=27 [YB-M: n= 25; SM: n= 2]). These informal conversations clarified the contribution of different job roles in keeping the hospital free from airborne disease transmission. A draft summary of barriers and enablers identified through the PAG, national leaders’ workshop, and preliminary contacts in hospitals is provided in Multimedia Appendix 1, which is the focus of a separate publication reporting on the development of an implementation strategy for rollout. Further description of the data collection process followed for wider stakeholder engagement is provided in Multimedia Appendix 2.

### Ethnographic Observations and Semistructured Interviews

Hospital staff were recruited following a combination of purposive and snowball sampling techniques. Participants in diverse roles (eg, infection prevention and control [IPC], estates, and clinical) were invited to capture the views of all those likely to be involved in the implementation of Air Safety. Potential participants were approached up to 3 times by email. We completed ethnographic observations in the work environment and online semistructured interviews following a predefined internal study protocol. Researchers had no prior relationship with participants. Participants had read a participant information sheet and knew the aims of the study. The researcher introduced themselves and their role in the study and explained the aims of the semistructured interview or ethnographic observation.

Ethnographic observations were used to understand hospital staff’s day-to-day involvement in keeping the hospital air free from infection while complementing and facilitating understanding of the content of interviews. Ethnographic observations were unstructured, but a minimal data collection template was used (Multimedia Appendix 3). These were conducted by female researchers trained in ethnographic research methods and with previous experience in ethnography (FS and YB-M). Hospital staff at both site 1 and site 2 (the two trial sites) were invited to participate in ethnographic observations; however, volunteers were only found for site 1. A total of 8 hours of ethnographic observation were conducted at site 1 with the 4 research participants who volunteered to be shadowed. Field notes were taken in situ and transcribed electronically as soon as possible after the ethnographic observation. Interactions with nonparticipants were not included in field notes.

Each semistructured interview was guided by an interview schedule (Multimedia Appendix 4) which had been pilot-tested in preliminary conversations with stakeholders. Two researchers with expertise in qualitative methods (YB-M and SEC) and a background in psychology and molecular medicine conducted the interviews (YB-M: n=9; SEC: n=9). The interviews were conducted iteratively, building on previous insights.

Semistructured interviews lasted on average 33.9 (SD 6.61) minutes (ranging from 25 to 49 minutes; median 33.09, IQR 28.7-38.1 minutes). All interviews were audio- or video-recorded on Microsoft Teams with automatic transcription. The researchers (YB-M and SEC) cross-checked transcripts for accuracy and pseudonymized the data. Given hospital staff’s limited availability, interviews were not returned to participants to check their content, but during the interview, summarization and clarification were used instead.

Semistructured interviews concluded when thematic sufficiency had been reached, considering input both from semistructured interviews and previous informal conversations with hospital staff from the same hospitals. No interviews were repeated. Participants did not provide feedback on the findings.

### Analysis

Ethnographic observations and semistructured interview transcripts were analyzed using codebook thematic analysis [21], which sits between coding reliability and reflexive approaches of thematic analysis [22], in NVivo (Lumivero; version 12) [23]. YB-M coded 2 interviews and developed a preliminary coding framework inductively that was presented and discussed with FS. The rest of the ethnographic observations and semistructured interviews, and the wider stakeholder engagement work, were coded by YB-M applying this framework. Coding was conducted iteratively, and new codes and refinements were incorporated and discussed with the team while analyzing the remaining data. Subthemes were organized according to the core constructs of NPT [16,17,24]. We selected NPT to understand how the use of AISaT would need to be embedded in the day-to-day routines within the hospital, rather than focusing on a list of elements that could influence its successful implementation. However, we have used the Consolidated Framework for Implementation Research (CFIR) in a separate publication to develop the implementation strategy for rollout and the Non-Adoption, Abandonment, Scale-Up, Spread, and Sustainability (NASSS) framework to understand how AI implementation was affected by different factors in previous research [19].

We completed the following steps: (1) familiarization with the data started during data collection and continued through successive readings of the transcripts and field notes; (2) generating initial codes involved inductive coding of the data; (3) refining the codebook led to the organization of codes into units of meaning, still quite attached to the data and similar to what would have been topic summaries; and (4) the NPT constructs were then brought into the analysis and initial codes were reexamined, searching for more interpretive content relevant for each of the constructs.

Different data sources were analyzed together, adding depth to the analysis. Summaries of data from all informal conversations, PAG meetings, conversations, and workshops with national leaders were aggregated with an indication of the respondents supporting it. Feedback from wider stakeholder engagement was initially analyzed separately, leading to the generation of summaries and informing recruitment and topic guide development for ethnographic observations and semistructured interviews. For instance, informal conversations with stakeholders suggested different hospital staff needed to be involved in deciding on the use of AISaT. Ethnographic observations suggested that those staff would be part of committees that would review and decide whether changes or the introduction of AISaT would go ahead. Subsequent semistructured interviews tried to unpack who would be leading the implementation and cascading those decisions at the local level. The aim of this study was not to quantify responses; instead, we have indicated within the findings section the agreement or disagreement between data sources, or the origin of the data supporting the different themes.

### Rigor

The workshop with national leaders was recorded to facilitate accurate note-taking by researchers (YB-M and SM) and support credibility. Likewise, semistructured interviews were recorded and transcribed to capture hospital staff’s accounts accurately. Field notes were taken during each follow-up conversation with national leaders and informal conversations with hospital staff. Field notes were then summarized via a Rapid Research Evaluation and Appraisal Lab (RREAL) data collection sheet [25] that reflected areas of agreement and disagreement, as well as areas requiring further data collection. Informal conversations were not audio-recorded as they were exploratory and aimed to understand the level of involvement of different professionals and their initial reactions. Once relevant staff had been identified through informal conversations and the study had received ethical approval, semistructured interviews were conducted with further staff in relevant roles to avoid participant burden, enrich data collection, and facilitate confirmatory triangulation. This approach, together with the ethnographic observations, allowed a more diverse and prolonged engagement of the researcher with hospital staff in relevant roles. The resulting sample size of the interviews was smaller but more relevant than those involved in informal conversations. To reinforce dependability, we kept a record of every interaction with stakeholders in a spreadsheet, field notes were made during informal conversations and ethnographic observations, and interview transcripts were checked and stored together to facilitate an audit trail. An entry was added to a reflexive diary after every interaction with the stakeholders, regular discussions were held with the rest of the research team, and quotes from interviews are provided throughout the findings to ease confirmability. Finally, while the aim of this study was not to produce generalizable findings but to inform the development of AISaT and the next stages of the trial, we described in detail the types of data collected and the setting, so other researchers can gauge the contextual similarities.

The implementation research team consists of experts in digital health, implementation science, medical sociology, and qualitative research. Researchers involved in data collection and analysis were not involved in AISaT’s development other than by providing our insights to members of the wider team, including engineers working on the development of AISaT. Hence, our knowledge of AISaT was limited to the information shared with participants and discussed during interviews.

### Ethical Considerations

The Air Safety Trial was approved by the Health Research Authority and Health and Care Research Wales Ethics Committee (ref 25/EM/0156). All participants involved in ethnographic observations and semistructured interviews provided informed written consent.

## Results

### Stakeholder Characteristics

We involved 12 public contributors (through the PAG) living around the Greater London area. All have a long-term condition and experience of care in multiple hospitals. Contributors were aged between 20 and 80 years, included 8 women and 4 men, self-identifying as Black, African, Caribbean, or Black British (n=4); Asian or Asian British (n=4); and White (n=4).

A total of 58 stakeholders were involved in conversations around the project and its potential implementation: 23 were involved in a workshop with national leaders (including senior NHS staff and policymakers, industry experts, and academics), and 8 national leaders took part in follow-up conversations, such as the 27 hospital staff who were identified either using online searches or purposive sampling within the 2 clinical trial hospitals.

Ethnographic observations were conducted with 4 hospital staff and semistructured interviews with 18 staff members, including 11 male and 11 female staff from Black, African, Caribbean or Black British (n=2), Asian or Asian British (n=8), and White (n=12) ethnic backgrounds. Staff interviewed had not been observed. Of 56 hospital staff members initially approached for observation and interviews at site 1, 5 declined to participate (feeling other colleagues were better placed to respond [n=1], having no time [n=4]). A further 22 did not respond to our contact, and with another 7, communication was lost after having expressed initial interest.

The job roles of those observed or interviewed were clinical (n=11), IPC (n=3), estates (n=6), and roles within the management of the hospital (n=2). Among those interviewed who shared job-related details, they had been in post for an average of 10.4 (SD 7.32) years (min=1; max=26), and most ranked the importance of ventilation within their roles as high (min 1 – max 10): 1 or 2 (n=1), 4 (n=1), 8 (n=3), and 10 (n=4).

Hospital staff interviewed or involved in follow-up conversations as part of the wider stakeholder work described several tasks undertaken as part of their role to reduce airborne disease transmission (summarized in Table 1). Their main role was isolating or managing patients with known airborne-transmitted infections, placing them in appropriate spaces (according to their risk level), or advising others on how to do this.

All stakeholders and participants provided their views on the hypothetical implementation of AISaT rather than during or after interacting with it. This work informed further development of AISaT prior to its testing and rollout.

**Table 1.** Hospital staff’s reported roles in keeping the hospital free from airborne disease transmission.

Category	Specific tasks
Room usage and air changes	Assessing room-use changes Checking that required air changes per hour are being achieved within rooms
Ward and patients’ oversight	Management and placement of patients with known airborne-transmitted infections (including isolation prioritization) Conducting ward audits Daily review of side-room usage Ensuring adequate testing of patients
Staff supervision	Ensuring staff are aware of infections and alerting IPC^a^ when needed Ensuring compliance with IPC training and practices Ensuring staff have adequate protection and providing related advice
Ventilation system management	Ensuring the ventilation system is functioning adequately Following Health Building Notes and Health Technical Memoranda
Liaison with other teams	Continuous liaison with estates regarding ventilation issues Attending multidisciplinary quarterly ventilation meetings within the trust

a IPC: infection prevention and control.

### Main Findings

Findings are organized according to the 4 constructs of NPT: coherence, cognitive participation, collective action, and reflexive monitoring (Table 2).

**Table 2.** Summary table.

NPT^a^ construct	Focus	Subthemes	Key insights	Expected enablers	Anticipated risks and barriers
Coherence	Understanding how the intervention could work in the hospital environment	Current practices to mitigate airborne disease transmission Shared goals Anticipated barriers and facilitators to adoption of AISaT^b^ Anticipated requirements for AISaT’s implementation	Understanding of value varies Organizational staff are optimistic, while clinical and IPC^c^ staff are more skeptical or cautious	Use in outpatient areas User-friendly interface Inclusion in national guidelines Senior leadership buy-in	Conflicts with fixed ventilation systems Old NHS^d^ building infrastructure PPE^e^ and hand-washing seen as higher priority Focus on aerosol particle dispersion versus air changes
Cognitive participation	Developing interest in the implementation and enhancing its relevance	Decision-making and ownership of AISaT Content and design of AISaT Timing of AISaT’s implementation	Engagement depends on multidisciplinary buy-in and having designated “champions” to lead	Multidisciplinary decision-making Local champions to cascade information Potential (though risky) mandated use	Lack of staff agency or ownership Staff burnout and workload Financial constraints Not seen as a top priority in comparison with patient care
Collective action	Planning for the implementation and rollout	Identification of resources Generalizability and sustainability	Implementation is anticipated to be hampered by technical fragmentation and the cost of maintaining mitigation strategies	External contractors to manage tool (reduces staff workload) Integration with EHRs^f^	Costs of potential implementation and maintenance of mitigation strategies Restricted data access Expected fragmented AI tool use within trusts Lack of universal EHR systems
Reflexive monitoring	Planning for the evaluation and refinement	Providing key metrics and assurances Futureproofing AISaT	Success is expected to be facilitated by long-term evidence and expert validation of effectiveness	Expert validation Strong evidence from clinical trials	Lack of a clear plan for future refinement support Uncertainty regarding postproject sustainability

a NPT: normalization process theory.

b AISaT: Artificial Intelligence Air Safety Tool.

c IPC: infection prevention and control.

d NHS: National Health Service.

e PPE: personal protective equipment.

f EHR: electronic health record.

### Coherence (How Do Stakeholders Understand AISaT and Its Recommended Mitigation Strategies?)

#### Current Practices to Mitigate Airborne Disease Transmission

The most common mitigation strategy used is the building management system (or air handling units) that represents the centralized ventilation system of the building. Our ethnographic observations captured how different units provide clean air and extract used air from hospital areas. Typically, the placement of ventilation outlets is decided by architects, and any changes to these require an authorizing engineer’s sign-off.

*HEPA filters available [within ventilation outlets] all have been allocated at design stage. It could be possible to uninstall as needed, things can change, and more things can be added. The authorising engineer (AE) for ventilation would need to agree and sign off*.

[Ethnographic observations, informal conversation, estates, HS104]

However, some interviewees raised concerns where portable HEPA filters have been trialed within the trust, mainly related to space constraints where they can obstruct staff’s work or pose a hazard to patients.

*if they [agitated patients] move the locker or they’re able to reach that and push it, it poses quite a danger of the actual physical unit being dropped and damaged, but also it then causing harm to somebody if it falls on them*.

[Semistructured interview, clinical, HS102]

Further concerns included the impact of a device, such as a portable HEPA filter, increasing room temperature and noise; competing for limited sockets; lacking evidence proving their effectiveness; taking significant time to clean the air; requiring maintenance plans and a large number of devices in wards; and their associated costs. Despite these reservations, staff who had HEPA devices within their work environment agreed they would be happy to have HEPA devices affixed to walls.

Other mitigation strategies reported included: prioritizing isolation of potentially infectious individuals, faster patient testing, improving hand hygiene, reducing the number of visitors when necessary, using personal protective equipment (PPE), constructing further isolation single or side rooms in new buildings, allowing longer cleaning times after conducting procedures with patients with certain infections, and using curtains or transparent partitioning screens. An interviewee flagged that some of this equipment is hardly being used after the COVID-19 pandemic due to limited storage and risk of falls for patients though. Also discussed in the interviews was that some spaces contain or have tested air filtration machines (such as HEPA mobile devices); however, this is not a generalized approach across the hospital.

*As far as I know there are some units like I think dental units that have filtration machines. But it’s not something that we would routinely put in all...areas such depends... on the risk assessment of what sort of procedure they’re going to undertake in the room, the size of the room, obviously. What’s the current air change? What’s the supply and extract...within the room. So yeah, we have some mitigations like filtration machines, but they’re not widely used...*

[Semistructured interview, IPC, HS110]

The NHS estate is generally old and comes with its own limitations (eg, lack of ceiling space, limited ability for new infrastructure projects, and limited electrical supply and clinical space).

*...when we build new buildings (...) there’s always more and more required from an engineering point of view. (...) you end up with a situation that (...) there’s just no space. (...) a good idea is to look at the ceiling heights and make sure that you’ve got more space. The problem is, the more space you have and the higher the building, the more expensive it is...*

[Semistructured interview, management, HS101]

Natural ventilation was not generally recommended but does seem to be the only option in some older buildings. Despite this, some staff in the older buildings had to work with sealed windows due to nearby building works.

Overall, mitigation strategies were welcomed as a way of protecting staff and patients, and staff were happy with current strategies in place but did share that they would appreciate further isolating rooms and “20 air changes that would be great. (...) that was the envy that I held for other areas. But in terms of [wanting further] technology, not really*”* (semistructured interview, clinical, HS103).

Hospital staff also reflected on the high costs of enhancing ventilation, the limited impact of mitigation strategies on air changes, and the limited number of isolation rooms. Due to financial constraints and the need for space, sometimes hospital areas have been repurposed without following the required procedures, resulting in tension between teams.

*...they’ll just change the use of a room, but obviously there’s a process to go through to do that, to make sure it’s safe, but not everybody understands that, and to be honest, they think (...) it’s a bit of a fuss, and they know it’s going to cost them money (...) And if they don’t have any money, but yet they desperately need to do something in a space because it’s the only space they’ve got, they’re just going to go ahead and do it*.

[Semistructured interview, management, HS101]

#### Shared Goals

Hospital staff interviewed reported that the aims of AISaT were a good fit with the trust’s overall goals of keeping staff and patients safe. Individuals involved in the wider engagement work raised that hospitals are perceived as risky spaces where people can catch infections, so they need to be protected, prioritizing the most vulnerable. Staff interviewed also described that the AI tool could be a source of reassurance to demonstrate the effectiveness of existing practices and support faster decision-making.

*I think it would give everyone reassurance. Certainly, in terms of pandemic or significant heightened risk, it would be helpful, not really for the confidence of the staff, but also the safety of the staff. And it would overcome the challenge we have in not being able to change the footprint of the healthcare facility*.

[Semistructured interview, clinical, HS103]

Interviewees were positive about the potential impact of AISaT on their work, as it would make their environment safer, if it did not take a lot of their time to input the data into AISaT. A couple of staff raised that AISaT could guide hospital staff but also architects and engineers to keep the hospital air clean and optimize staff capacity and space use.

*If we had a tool like that (...) every time that you had a new space (...) you could run the whole of the design through the AI software, and it would identify where you may (...) add in things at the base build level (...) I can’t see a problem with using models to help to help give you the optimum design for a room. It’s a good idea*.

[Semistructured interview, management, HS101]

#### Anticipated Barriers and Facilitators to Adoption of AISaT

Hospital staff observed and interviewed, and wider stakeholders, pointed out that new buildings still have ventilation issues, but that it may be easier to implement AISaT in these settings as they are more likely to have standardized room measurements to comply with the latest regulations. Hospital staff also raised that AISaT would be particularly valued in areas where ventilation cannot be improved systemically across the site (eg, old buildings).

*...in an ideal world, we would have something like a built-in air conditioning system or something that was systemic across the building. But being such an old building and I think the electrical grid is the reason why we couldn’t have anything more systemic built-in. So, we’re having to rely on things like windows and maintaining good infection control with our isolation in our side rooms...*

[Semistructured interview, clinical, HS102]

Concerns related to staff resistance were raised as:

it is quite tricky in a Trust to get them [staff] to accept changes like this and to get them to think it’s worthwhile because they can’t. They don’t necessarily see the value of it.

[Semistructured interview, management, HS101]

Not seeing a clear benefit from AISaT and its recommended mitigation strategies or not experiencing an immediate benefit were anticipated to be the main barriers to staff engagement. Additional anticipated barriers included the lack of awareness and knowledge around ventilation and the impact that the positioning of mitigation strategies can have; limited digital understanding and guidance; lack of preventative mindset; and limited trust or confidence in AI in general. In contrast, some hospital staff felt the pandemic had raised staff’s awareness of ventilation, and they were now actively requiring ventilation improvements within the trust. Trust in AI was also described as a potential facilitator for the adoption of AISaT, as staff reported AI is becoming more present in daily lives and at work. Hospital staff’s workload could be a source of staff resistance; however, if the AI could reduce workload, it could be seen also as a facilitator.

Some hospital staff interviewed and involved in wider engagement work referred to inpatient areas or wards as areas where the potential implementation would not be a priority given the number of people working within those areas, with the associated movement, the lack of space, connectivity issues within hospitals, and the number of beds which would require several HEPA devices. Wider stakeholder engagement work involving hospital staff encouraged the use of mitigation strategies already existing within the wards, and that AISaT should consider the space available and size of any new suggested strategies. The potential implementation of AISaT in outpatient areas, isolation rooms, labs, or neonatal spaces was more acceptable.

*...you have 6‐7 patients in there and then some patients, they bring quite a lot of their stuff...they put a lot of things in their cupboard and we have only a small one* (...) they need to consider... space (...) *It’s another tool [HEPA device] that they don’t see any results from it. It’s just there physically. (...) they’re still getting sick. They’re still getting infection (...) I don’t think anything would eradicate that. They don’t see any success to it. They’re just thinking, you know, it’s another thing that’s occupying the space of the patient, that it’s unnecessary. It’s another tool that...they have to make sure it’s on (...) let’s be honest, it’s not a priority for us to do that. It’s not our job...to do that (...) it shouldn’t be us who needs to check it*.

[Semistructured interview, clinical, HS108]

Besides some clinicians not feeling that dealing with AISaT and the associated mitigation strategies would be part of their role, an interviewee was struggling to think in what area of the hospital the AI tool could be of use:

*...[AISaT] it’s an interesting tool... in certain areas like theaters or operating rooms I don’t think they would be useful because if the air changes... will have to depend on the... centralized ventilation system, because...they’re essentially critical departments...With the AI tool, I’m not sure where we can use them*.

[Semistructured interview, IPC, HS110]

Stakeholders mentioned that PPE may be seen as a cheaper alternative to the mitigation strategies AISaT may offer. This was in contrast with several staff members mentioning the inconvenience of working with PPE: “It’s not amazing, to be honest, if you can imagine doing quite a difficult procedure over a pronounced amount of time wearing masks and PPE is not amazing.” (semistructured interview, clinical, HS112), particularly in buildings with poorly balanced temperatures.

Hospital staff did not perceive the need for further mitigation strategies in their workplace, and some failed to see the point of AISaT. However, highlighting AISaT advantages, such as staff and patient benefit and cost reductions, may be needed to make AISaT attractive to hospital staff (see the “Reflexive Monitoring” section).

*People’s time is the crux. (...) you can usually win hearts and minds from people if they can really see a benefit to a bit of effort that they might have to put in during implementation. But if they can see that this is going to reduce my staff sickness is going to mean I’ve got less patients getting cross infected on the wards. And I’m going to have my beds all open and not closed due to infection. I think they’re massive pluses*.

[Semistructured interview, management, HS111]

#### Anticipated Requirements for AISaT’s Implementation

Hospital staff and wider stakeholders suggested using a series of strategies that could facilitate implementation and a future rollout of AISaT. The most frequently discussed strategy was gaining buy-in from senior staff (eg, the chief executive, IPC director, and authorizing engineers), board members, and champions on the ground.

*Your best bet is to go to the director of infection control and prevention. If he was interested and if he thought it was an advantage, I would say that would be a good place to start. (...) If you could get [Chief Executive] interested, obviously that would be the best person (...) if he was interested and thought it was a good idea and it was protecting his patients*.

[Semistructured interview, management, HS101]

Clear communication was recommended, with staff and patients, about what is needed from users and about AISaT complementing human decision-making. Also raised was the need to transparently report how AISaT is better than a human, how the AI works, and clarity on its benefits as well as its limitations.

Wider stakeholder work suggested that gaining support from recognized bodies would also be required, through appearance in guidelines such as NICE (National Institute for Health and Care Excellence); dissemination at national meetings (National Clinical Engineering, Ventilation Teams Meetings); and having the support of medical organizations.

Providing training on AISaT to hospital staff who would use it or work around it could facilitate its uptake and build trust in the AI tool. Ensuring all staff are involved in training could be positive, to promote inclusivity and facilitate the acceptability of AISaT. Stakeholders thought training should include ventilation, the relevance of positioning the mitigation strategies, and AISaT itself. However, one interviewee doubted the need for training as they envisaged the implementation of AISaT as a one-off interaction with an external contractor. Another participant cautioned that colleagues may not react well to training on ventilation when they are faced with poor ventilation levels, high temperatures, and are unable to open (sealed) windows or use fans due to electrical supply limitations and scarcity of sockets.

*I think it [training] would be beneficial, but I think the response would be...interesting. (...) we’ve had such challenges every summer...where it’s been very hot on the ward and we’ve had to use fans and...We’re trying to as best as we can to isolate these patients who’ve got airborne infections to then talk about ventilation and the joys of ventilation. I think that may not be received as well*.

[Semistructured interview, clinical, HS102]

Hospital staff were generally cautious and expressed concerns about the need for appropriate validation and assurances that the AI would be providing safe recommendations. This caution was not necessarily negative, as “proper use of it could really help ease our workflow (...) having it do the recommendations for you and then you just check it makes it convenient” (semistructured interview, IPC, HS113). However, some interviewees stated they could not comment on the acceptability and potential to implement AISaT without having seen it, and knowing in detail how it had been trained, or having a summary of its impact when used within the hospital environment.

### Cognitive Participation (How Will Staff Engage With AISaT and Its Recommended Mitigation Strategies?)

#### Decision-Making and Ownership of AISaT

It was agreed that early staff engagement would be required, not only to gather their views to develop AISaT but to facilitate the decision-making process of who should take ownership of the AI tool. Like current practice, the development of a multidisciplinary working group would ensure the decision-making was not “down to a person” but to a multidisciplinary team to determine whether AISaT should be used, and the details on when and how.

First funding would need to be sought. Then, the contractor (HS104 and their team) would gather evidence and information about AISaT. Then they would present the idea to the Committee. If the Committee agrees, then they would need to ensure buy-in from IPC. Discussions between estates, medical, and IPC teams would need to happen to explore what effect this is going to have (eg, having to close an area to assess the impact of adding new HEPA filters or placing them where AISaT suggests) and would provide suggestions as to how this effect could be managed. Nine out of 10 times medical teams and IPC will say no; the area cannot be closed, and there will need to be negotiations until there is agreement between all parties. This may mean that the implementation does not occur soon, as pointed out during ethnographic observations.

A few interviewees suggested a similar process of group decision-making when choosing among the recommended mitigation strategies suggested by AISaT.

*...the decision will not ultimately be from one person. It’s always a multidisciplinary thing.You’ll have to present the options to the end users, to other experts to see what is really the best option in practise*.

[Semistructured interview, IPC, HS110]

*Say it’s showing that we need HEPA filters... 100% estates, finance, infection control would all want to be involved in that. And it wouldn’t be...just down to the ward manager to say, yeah, we’re going to buy four HEPA filters for this ward.*

[Semistructured interview, clinical, HS203]

Another interviewee suggested that the implementation of AISaT should come as an imposition from IPC rather than an exercise requiring hospital staff’s engagement and acceptance.

*I think for this realistically it wouldn’t so much be staff engagement and encouragement, it would be imposed. So, if it’s supported, it would be supported on one ground or another, be a patient safety or financial and they would be imposed*.

[Semistructured interview, clinical, HS103]

On this line, another interviewee wondered whether the strategies shared by AISaT would be recommendations (something staff could choose to follow or not) or actual policy they would need to follow. If the latter, current policies in this area come from the trust and from IPC, so any AISaT recommendations should be aligned with those existing policies.

Despite most interviewees being in favor of a multidisciplinary effort involving IPC, clinical, estates, and operations for AISaT’s implementation, none of them suggested themselves or their team would be involved in leading the implementation of a tool such as AISaT. Only one IPC staff member agreed when prompted by the researcher that IPC may be best positioned to lead on AISaT’s implementation.

*Well, it could fall under IPC... 'cause sometimes with estates, if we let them lead on it...some trusts outsource them. So, I do get the point of IPC trying to lead on this... I would agree, it’s part of the things that we check and that we try to enforce as a team to ensure that the ventilation throughout the hospital is good. So yeah, I think we can take a lead on it*.

[Semistructured interview, IPC, HS113]

Most hospital staff involved in interviews and wider stakeholder work placed ownership on IPC: “infection control [IPC] (...) can oversee the project because (...) when it comes to infection, they need to be involved in that*”* (semistructured interview, clinical, HS109), given their cross-trust presence. Contrasting this, an IPC staff member insisted this would be a role for the estates team, who have expertise in ventilation and lead critical ventilation meetings.

*I think it should be a multidisciplinary approach on the initial stages until we all have a full understanding of...how it works in practise. But I think ultimately it will have to be estates leading on it. Obviously with our...expert input from ...clinical areas, IPC, ventilation engineers...Anything ventilation related would fall under estates responsibility*.

[Semistructured interview, IPC, HS110]

*I think the IPC would probably come to someone in estates and we would accompany them to that space, and if there’s a measurement issue, we could figure that out... But they [IPC] would decide where is the best place to put any device that might impact have a clinical impact on patients or staff.*

[Semistructured interview, estates, HS116]

There were mixed opinions among hospital staff involved in interviews and wider stakeholder work who felt that, as long as staffing costs were funded, then the inputting of data into AISaT could fall under estates, engineers, or IPC.

There was more agreement in the cascading down of decisions where estates and facilities teams would be the ones in charge of cleaning, maintaining, and replacing the recommended devices or equipment to mitigate airborne transmission of infections. Interviewees further raised that if the HEPA filters are considered nonmedical devices, then installation could come from estates.

#### Content and Design of AISaT

There was a difference in opinions on how AISaT should be designed to facilitate its uptake. Some hospital staff thought a ranking of potential mitigation strategies that are the best at preventing airborne transmission of infection should be provided. They felt that, regardless of the lack of availability within the trust, staff having a comprehensive list may help the trust push to purchase these items, especially in preparation for a future pandemic. Others suggested a ranked list should only contain mitigation strategies available within the trust as it may create anxiety among staff if they do not have access to the best rated options, or they may get “overwhelmed” (HS113) by a list of endless options.

Interviewees shared some technical factors that may help with the implementation of AISaT; this included ease of use, integrating AISaT within NHS systems (or whitelisting websites or the AI tool); compatibility with diverse devices such as computers and iPads; facilitation of automatic data entry through LiDAR software to capture room dimensions, or importing patients’ data from electronic health records. Further suggestions on AISaT’s functionality included its ability to perform a risk assessment for the use of physical separation equipment and an air filter (eg, HEPA device) with a prediction model to alert staff of outbreak risks. Also suggested was demonstrating through AISaT the variable cost of a mitigation strategy, which could help to choose from what is available in the market. The ethnographic work suggested it would be helpful if AISaT could keep a record of HEPA filter performance and dates of installation, and other stakeholders suggested AISaT could include reminders to staff to attend PPE fit testing. It was suggested that AISaT should account for movement and that it should consider the fact that staff may be contagious. This is particularly relevant in wards with more vulnerable patients who are usually seen by a range of staff. Two clinical hospital staff members also reflected on the need to involve clinical experts to tailor the AI tool to the needs of different areas with different ventilation needs.

*...you need people or clinical staff that understand their local areas so you can tailor it for the area that you’re using it in because each procedure each diagnosis, medical diagnosis, etcetera has a different outcome for every different area*

[Semistructured interview, clinical, HS112]

#### Timing of AISaT Implementation

Among interviewees, many agreed that implementation of AISaT was not a priority now, but it would become a priority in future pandemics and, hence, highlighted the relevance of this research. However, about one-third of those interviewed felt AISaT is currently relevant for the prevention of infection.

*I’d hope that you could use it every day, because I’d hope it would really help us to prevent cross infection, prevent outbreaks...So I think... for prevention, be proactively using it... I’m sure it’s useful...in a pandemic or an outbreak as well. But...I’d want to use it before you got to that point. I’m completely with...try to prevent it and control it*.

[Semistructured interview, management, HS111]

### Collective Action (How Will Staff Work to Implement AISaT and Its Recommended Mitigation Strategies?)

#### Identification of Resources

Often, hospital staff were unclear about who would fund the potential implementation and rollout of AISaT and its recommendations. When hospital staff had suggestions, they mentioned:

*...maybe NHSE can provide a sponsorship if we’re looking into a bigger perspective of the whole UK.*

[Semistructured interview, clinical, HS109]

However, there were also suggestions that “executives would agree to fund from a central pot because it’s not one area (semistructured interview, management, HS111)” or that Integrated Care Boards (ICBs) should be able to provide funding or suggestions of possible funding. The need to develop a business plan to cost staff and time required for the implementation of AISaT and its recommendations was mooted.

Likewise, hospital staff interviewed and involved in wider stakeholder work raised concerns about the costs to maintain the mitigation strategies (eg, HEPA filters), as well as the impact of maintenance on staff’s workload and capacity. They also referred to the high costs of making changes to ventilation systems and that any changes must meet the latest guidelines or Health Technical Memoranda (HTMs). Conversations reflected the items’ costs (eg, AISaT and implementing its recommendations), the associated staff costs (eg, data entry into the AI tool, delivering on maintenance plans, and so on), and concerns as to whether the trust would be willing to invest in AISaT and its recommended mitigation strategies even if the trial proved its effectiveness.

*I think with the amount of (...) maintenance involved like routine cleaning, routine maintenance, reporting of equipment that’s faulty, it’s already a bit of a challenge just on day-to-day with clinical items that patients use(...) It can be quite challenging on a large [busy] ward (...) and a substantial service to then add another item in there that isn’t immediately impacting the day-to-day tasks or benefiting the patients in that immediately visible way. It can be a barrier*.

[Semistructured interview, clinical, HS102]

As previously mentioned, an interviewee envisaged AISaT being used by an external contractor, as the rooms of interest would usually maintain their size and characteristics, and so the need to enter data into AISaT and to choose a recommended mitigation strategy was perceived as a one-off action. However, the one-off use was shared by most interviewed: “you probably only use it once, and that’s probably it (semistructured interview, clinical, HS202),” who would only use it again if there was a change in the placement of objects in the room. Such implementation was seen as advantageous as it would not impact staff’s capacity or require further training. In successive interviews, further hospital staff members agreed this could be a feasible option to facilitate implementation without negatively impacting staff’s workload.

*...Like a contractor. (...) Yeah, like I said... it’s just another thing to think of and we already have a load of things...you know, pressured. So you...don’t want to commit to something that you can’t really [do]*.

[Semistructured interview, clinical, HS108]

#### Generalizability and Sustainability

Hospital staff interviewed raised that communication among staff could become a barrier to the implementation of AISaT when different members of staff work on similar issues but from different teams, such as the NHS and contractors working together. Also raised was fragmentation in how different trusts incorporate new devices, and a lack of guidelines on how to integrate new digital developments into hospitals, which would take a long time to develop. Unsurprisingly, the acquisition of funding for AISaT’s implementation may also be different across trusts.

Access restrictions were also identified as barriers by hospital staff involved in wider stakeholder work regarding data on room characteristics (eg, size, height, objects in the room, and ventilation characteristics) and floor maps. Difficulties may also arise when attempting to import data, as floor maps are often in PDF format instead of in electronic formats that could be easily exported. Different hospital buildings, even within the same trust, also have different levels of data available within their records. This would, therefore, require manual data entry, which would require costing staff time.

The ethnographic work suggested that the potential implementation of AISaT and its recommended mitigation strategies would need approval from multidisciplinary committees. The implementation timing would need to be agreed, as it is not uncommon for projects to be approved but face delays in green lights for actual implementation. Within the approval process, the different management systems in different buildings (eg, privately funded or NHS-maintained) would need to be factored in, as there are differences in these processes even for buildings within the same trust. In privately funded buildings, for instance, there is the need to factor in the landlord’s position regarding the implementation of AISaT and its mitigation strategies.

*Don’t forget that this building is not owned by the NHS. It’s rented. They [Landlords] would need to be in agreement with it, so you’d have to bring them in if potentially you were thinking about that [installation of mitigation strategies]*.

[Semistructured interview, clinical, HS112]

### Reflexive Monitoring (How Will People Assess the Success of AISaT and Its Recommended Mitigation Strategies?)

#### Providing Key Metrics and Assurances

Hospital staff interviewed referred to the need to prove the effectiveness of AISaT, addressing the 3 NHS priorities: costs, effectiveness, and implementation practicalities. Key metrics should include those of interest for IPC, such as the extent AISaT reduces infections; whether it has an impact on staff, patients, or both; whether it leads to bed-day savings or fewer closed beds, fewer appointment cancelations (in outpatients); and reduction of outbreaks. But also the extent to which it is effective, such as the impact on air changes per hour; its energy and financial savings (eg, reduction of costs to ventilate an area). It would need to produce benefits on safety grounds but also need to lead to financial benefits.

*...they’re pushing for sustainability and they’re pushing for capacity, bed capacity, patients flow and reduction of infections as such. And it would help the hospital if it’s done properly, that they have seen all the areas of concerns before they actually invest into something. Because...we’ve seen some projects that had been invested, they’ve spent quite a lot of money, but it hasn’t been pushed through...before the commitment to be set out there, they really need to make sure that this is... worth it to the benefit of the hospital, the patients and the staff*.

[Semistructured interview, clinical, HS108]

Beyond the health economics assessment, hospital staff also anticipated the need for expert validation to explore to what extent AISaT’s recommendations are aligned with gold standards, and evaluate differences between human and AISaT, to explain why AISaT is better.

*As long as we’re happy that it’s...tested and validated and that there’s always the double check until we’ve got confidence that it does actually predict. I think there’s got to be that validation process continually (...) sort of accuracy tolerances built in. (...) Because it’s there’s a lot at stake if it takes us to the wrong decision or the wrong action*.

[Semistructured interview, management, HS111]

Participants also suggested the need for live monitoring of the quality of the air in parallel to the implementation of AISaT and its recommended mitigation strategies.

*If introducing HEPA, there would need to be a live measurement of pressure, as this will tell them when the filter is filled and needs changing*.

[Ethnographic observations, notes from informal conversation, estates, HS106 and HS107]

#### Future-Proofing AISaT

Interviews with hospital staff and conversations with those involved in wider stakeholder engagement referred to the need to develop AISaT to be able to adapt to different technologies (eg, PC, phones, tablets, and advancements in these devices), settings (eg, different hospitals and room characteristics), and policy, as these are likely to evolve with time. When considering preparedness for the future, an individual involved in the wider stakeholder engagement work raised the need to confirm who would be responsible for refining and providing technical support to hospitals following the research trial or development phase of AISaT, showing concerns about the ongoing feasibility and ownership of AISaT.

## Discussion

### Principal Findings

This paper explores key stakeholders’ anticipated barriers and facilitators to the implementation of AISaT to improve its acceptability at the implementation stage [11]. Our findings, organized around NPT, suggest that while AISaT and its recommended mitigation strategies are generally positively received, their wide implementation may be hindered by its associated maintenance and staff costs; the lack of perceived need and immediately observable benefits; and organizational differences within trusts and hospital buildings.

### Coherence (How Do Staff Understand AISaT and Its Recommended Mitigation Strategies?)

Coherence was variable across hospital staff. While hospital staff in organizational roles anticipated AISaT’s usefulness in their daily work, hospital staff in clinical and IPC roles were more divided on the value of the AISaT tool, with some reluctant to share their views on AISaT’s potential prior to reviewing results from a clinical trial. This may reflect staff not seeing the clinical need required to facilitate AISaT’s implementation [26]. The main risks anticipated to AISaT’s implementation were that existing strategies used within hospitals, such as fixed ventilation systems, are hard to change and may be different from the strategies AISaT may recommend, which are likely to involve portable HEPA filters. Additional anticipated risks to AISaT’s implementation were the potential challenges associated with HEPA devices and the lack of standardized rooms in old NHS buildings. A number of additional potential challenges have also been identified, such as PPE being seen as a more economical alternative; that handwashing and isolation rooms are being prioritized in current guidelines [27]; and that hospital staff felt improving air changes should be prioritized, whereas AISaT’s current prioritization is focused on diminishing aerosol concentrations. The main expected enablers identified related to the implementation of AISaT in outpatient areas; ensuring the AISaT interface is easy to use and responsive to staff’s needs; buy-in from senior staff; and its dissemination through national guidelines. In line with findings from previous research, there is a need to communicate that using AISaT does not remove human involvement from the decision-making process [11,15]. AISaT may be more likely to enhance its coherence in the hospital environment by tailoring its implementation approach to the ways of functioning of different hospitals, while providing staff with proof of the effectiveness of the tool in a way that is relevant for the metrics used within the NHS (eg, justifying cost savings and benefits for staff and patients).

### Cognitive Participation (How Will Staff Engage With AISaT and Mitigation Strategies?)

Cognitive participation is anticipated to depend on multidisciplinary decision-making on the implementation of AISaT and its recommendations and having champions to cascade decisions down and encourage implementation on the ground. Anticipated barriers to implementation could be overcome by imposing the use of AISaT rather than relying on staff’s engagement. Imposition, however, would come with the inherent risk of losing the feeling of shared ownership, which would add to a lack of agency among staff, becoming a potential implementation challenge [28]. The potential implementation of AISaT was often not perceived as a priority, in a climate where staff feel overloaded, there are increasing financial constraints, and direct patient care is the most pressing matter. Such a lack of agency, particularly among those in clinical and estates roles, while raising the need to involve multidisciplinary teams may reflect the ways of working within the NHS as well as the nature of the AISaT tool falling in a confluence of responsibilities among different roles.

### Collective Action (How Will Staff Work to Implement AISaT and Recommended Mitigation Strategies?)

Collective action is anticipated to be impacted by costs associated with the implementation and maintenance of AISaT and its recommended mitigation strategies; the fragmented implementation of AI tools and devices within trusts; restrictions in accessing data; and variations in approval processes within trusts and buildings. While it was suggested that AISaT could be best integrated within a comprehensive electronic health record, this comes with an added layer of difficulty since electronic health record systems are not universally used across UK hospitals, which would require further adaptations to different systems used. This is a known generalizability issue previously identified in the literature [29,30]. An expected enabler identified was the external implementation of AISaT (eg, by a contractor), which would reduce its impact on staff workload.

### Reflexive Monitoring (How Will People Assess the Success of AISaT and Mitigation Strategies?)

Reflexive monitoring is anticipated to derive from expert validation and evidence on effectiveness and impact prior to the implementation of AISaT. However, previous research has also pointed out that such evaluation should continue after implementation [31]. An anticipated risk identified is the need for future refinement support that is unclear after the end of this project.

### Comparison With Prior Work

Our findings are consistent with those of a recent systematic review where AI implementation within hospitals was found to be influenced by understanding and trust in AI, quantification of the benefits of the AI, external validation to facilitate generalization, costs of implementing the AI, training staff and maintaining the AI, and the availability of policies to fund and regulate AI solutions [19]. However, our findings also point toward other critical elements, such as specific environmental characteristics where the AI and mitigation strategies will be implemented (eg, old buildings with busy and crowded wards, with scarce space and overloaded hospital staff), together with the priority given to AISaT and its mitigation strategies within the existing ways of working and measures already available. These findings also align well with a recent criticism of the NHS 10-year plan that AI implementation cannot sort out the whole structural issues within the NHS such as the constrained budgets [32]. Likewise, our findings are aligned with the need to transparently report on how the AI was trained as well as the benefits associated with its use, providing relevant metrics to the end users, and acknowledging the limitations of the tool [15].

Beyond our focus on AISaT’s implementation, but very closely related, difficulties in the use of HEPA devices, such as noise, which had been previously reported in the literature [33], did not preclude their hospital-wide use through ventilation outlets installed in ceilings. This is in line with HEPA filters being the most common mitigation strategy used in hospitals to reduce airborne disease transmission within papers included in a recent review [18], subsequent research [34], and aligned with estates technical guidelines [35]. Although the review reported improvements in patients’ health outcomes [18], some stakeholders included in our study stressed the lack of randomized controlled trials proving HEPA devices’ effectiveness.

### Future Research

This work involved extensive stakeholder engagement, capturing views from the public, subject matter experts, and hospital health care professionals, which helped identify common ground, but also areas of discordance between accounts even among professionals with similar job roles. Hence, it responds to the recommendation to include stakeholders at an early stage to facilitate future acceptability of AI tools [15]. This study is not without limitations; we struggled to access the hospital environment for further ethnographic work, only being able to observe estates work within one building. Difficulties may have been aggravated by the timing of our research during flu season. Furthermore, AISaT was under development at the time of conducting this study, and hence neither researchers nor participants had a tangible idea of what the tool would look like. This may have made it difficult for hospital staff and stakeholders to provide more specific responses and imagine what its implementation could look like in practice. Our formal data collection sample was focused on hospital staff from 2 hospitals, but more so from one of them, due to low response rates and delays in getting the relevant authorizations to do research in the second research site. Hospital staff shared their reservations about the AI tool during interviews; however, there may have been a natural selection bias. Through the wider stakeholder engagement (eg, via a national leaders’ workshop and follow-up conversations with national leaders), experts from around the country were involved, which helps reinforce the applicability of our findings to other hospitals with caution. Areas of concern such as impact on costs, workloads, and diverse ways of working were raised by all parties, supporting the transferability of our findings to other hospitals and health systems working under limited resources, staffing pressures, and in old buildings. This approach supported our ability to provide analytical generalization, to aid the development of the AI tool and future trial plans. Future research should involve relevant stakeholders in designing user-friendly AI tools and seeking their feedback with demonstrations of the product to visualize the end-product and think of ways to implement it in their practice.

### Conclusions

Even when AISaT and its recommended mitigation strategies are not considered a priority within the hospital environment now, in a climate of tight budgets, staff shortages, and heavy workloads, staff expressed their openness to accommodate AI innovations if the effectiveness and benefits are proven and shared with them. Therefore, when planning implementation across hospitals, it will be important to frame this according to the tenets of NPT to enable understanding at the level of the structure of services as well as people’s experiences to engender the flexibility that will be necessary to maximize the likelihood of success across different NHS trusts and associated hospitals.

## Supplementary material

10.2196/93420 Multimedia Appendix 1 Determinants of implementation of Artificial Intelligence Air Safety Tool identified through wider stakeholder engagement.

10.2196/93420 Multimedia Appendix 2 Detailed data collection process followed for stakeholder involvement.

10.2196/93420 Multimedia Appendix 3 Ethnographic observations sheet.

10.2196/93420 Multimedia Appendix 4 Semistructured interview topic guide.

10.2196/93420 Checklist 1 COREQ 32-item checklist.

## Abbreviations

AISaT: Artificial Intelligence Air Safety Tool
CFD: computational fluid dynamics
CFIR: Consolidated Framework for Implementation Research
COREQ: Consolidated criteria for Reporting Qualitative Research
HEPA: high-efficiency particulate air
HTM: Health Technical Memoranda
ICB: Integrated Care Board
IPC: infection prevention and control
NASSS: Non-Adoption, Abandonment, Scale-Up, Spread and Sustainability
NHS: National Health Service
NICE: National Institute for Health and Care Excellence
NIHR: National Institute for Health and Care Research
NPT: normalization process theory
PAG: patient advisory group
PPE: personal protective equipment
RREAL: Rapid Research Evaluation and Appraisal Lab
