# Supplementary material for: Anticipated Barriers and Facilitators to the Implementation of an AI Tool Advising on the Placement of Mitigation Strategies to Improve Air Quality in the Hospital Setting: Qualitative Study
Source: JMIR Hum Factors. 2026 Oct 13;13:e93420. doi: 10.2196/93420 (PMC13660969; doi:10.2196/93420)
Supplement: Supplementary file 1 [file humanfactors-v13-e93420-s002.docx]

|  | **Activity** | **Dates** | **Sample** | **Length** | **Topics discussed** |
| --- | --- | --- | --- | --- | --- |
| Wider stakeholder engagement | In-person Patient Advisory Group (PAG) meeting | 27/11/2024 | 12 | 2.30hrs  (afternoon) | - (Lack of) Relevance of the study. - Reactions to HEPA filters in hospitals (including placement preferences and concerns) - Reactions to AISaT’s concept (including what patients would like to know and what other things should be done in hospitals) - How well does AISaT solve the issue? |
|  | Online workshop with national leaders in ventilation, initial joint introduction of the project followed by two break out rooms for smaller group discussion. | 15/05/2025 | 23 | 1h  (lunch time) | - (Lack of) priority of airborne disease transmission prevention within hospitals. - Advantages and disadvantages of different measures used. - Reactions to AISaT’s concept and the use of AI to guide the prevention of airborne disease transmission. - Relevant roles for implementation within hospitals. - Relevant contacts to involve in subsequent conversations. |
|  | Follow-up conversations with national leaders | Between 19/06/2025 and 18/07/2025 | 8 | 30mins/conversation (adapted to availability) | - The Air Safety project. - Experience working on ventilation projects. - Expected barriers and facilitators to implementation. - Relevant contacts. |
|  | Informal conversations with hospital staff | Between 16/04/2025 and 08/09/2025 | 27 | 30mins/ conversation (adapted to availability) | - Role responsibilities and relevance of ventilation. - Initial reactions to mitigation strategies. - Initial reactions to the use of AI to guide the placement of mitigation strategies. - Hospital room characteristics. - Acceptability of data input process required by AISaT. - Barriers and facilitators to roll-out. |
| Formal data collection | Ethnographic observations | Between 15/10/2025 and 18/12/2025 | 4 | 8 hours 10minutes in total | - Shadowing staff conducting work on hospital ventilation. |
|  | Semi-structured interviews |  | 18 | On average 34 minutes (ranging 25 to 49 minutes, median 33.09 minutes) | - Role responsibilities, relevance of ventilation and difficulties encountered in reducing airborne disease transmission within the hospital. - Mitigation strategies acceptability. - Anticipated AISaT’s acceptability. - Anticipated AISaT’s barriers and facilitators to implementation. - Anticipated roles required to be involved in implementation. - Anticipated roll out feasibility and requirements. |
