# Supplementary material for: Anticipated Barriers and Facilitators to the Implementation of an AI Tool Advising on the Placement of Mitigation Strategies to Improve Air Quality in the Hospital Setting: Qualitative Study
Source: JMIR Hum Factors. 2026 Oct 13;13:e93420. doi: 10.2196/93420 (PMC13660969; doi:10.2196/93420)
Supplement: Supplementary file 2 [file humanfactors-v13-e93420-s001.doc]

| **Intervention Characteristics** | - User friendly, easy to use tool (Enabler, Preliminary conversations with <Site 1> staff) - Integrated system both for AI and mitigation strategies (ceilings, panels) (Enabler, PAG, Preliminary conversations with <Site 1> staff) - Adaptability to other settings and future mitigation strategies (Enabler, PAG) - Reduced staff data introduction/Staff time (Enabler, Preliminary conversations with <Site 1> staff) - Mitigation strategies requiring space (Barrier, PAG, Preliminary conversations with <Site 1> staff) - HEPA (reliant on electricity, noise, heat, unclear cleaning plan, malfunctions/fire risk, lots needed in large inpatient wards) (Barrier, PAG, Preliminary observations from service evaluation, Preliminary conversations with <Site 1> staff, Ward isolation solutions meeting observation, National leaders) - Fans noise complicate assessments of neurological patients (Barrier, Room Selection observation) - AI as complementary to human decision-making (Enabler, PAG, Preliminary conversations with <Site 1> staff) - Trust in AI as something more efficient and inevitable (Enabler, Preliminary conversations with <Site 1> staff) - Lack of trust in AI/digital understanding – reliable? safe? (Barrier, PAG, Preliminary conversations with <Site 1> staff) - Involving multidisciplinary work - difficulty identifying implementation leadership (Barrier, Preliminary conversations with <Site 1> staff) - Cost/Access to funding (Barrier, Preliminary conversations with <Site 1> staff) - Staff capacity (Barrier, Preliminary conversations with <Site 1> staff, National leaders) - Potential benefit for staff, patients and the Trust (Enabler, Preliminary conversations with <Site 1> staff) - Rating all possible options – more valuable but may lead to staff anxiety (Enabler and barrier, Preliminary conversations with <Site 1> staff, National Leaders) - AI Effectiveness (Enabler, Preliminary conversations with <Site 1> staff, Ventilation meeting notes Aug2025) - Face masks are often seen as cheaper and quicker than other alternatives (Barrier, Preliminary conversations with <Site 1> staff) |
| --- | --- |
|  |  |
| **Outer Setting** | - Patients and staff wellbeing at the heart of the NHS (Enabler, Preliminary conversations with <Site 1> staff, National Leaders) - NHS 10-year plan to digitalise/include AI and reduce staff sickness levels (Enabler, National Leaders) - Energy saving (Enabler, Preliminary conversations with <Site 1> staff having read study summary) - HTMs for recommended room sizes and air changes per hour (Enabler, Preliminary conversations with <Site 1> staff and National Leaders) - Very fragmented integration of devices within the NHS (Barrier, National leaders) - Differences between trusts (culture, team working, ICB strains) (Barrier, National leaders) - Lack of modern guidance to incorporate the digital within the NHS (Barrier, National leaders) |
|  |  |
| **Inner Setting** | - Good communication among staff and between staff and patients -e.g., aim and benefits of the intervention, not to swich off devices, etc. (Enabler, PAG) - Accessible communication campaign to inform patients (Enabler, PAG) - Old infrastructure: NHS buildings, lack of standard room measures, not much ceiling space/difficulty installing new things, difficult to regulate temperature, limited electrical capacity, lack of clinical and patient space, without windows or ones that cannot be opened, not a lot of natural ventilation. Some newer buildings still have ventilation issues. (Barrier PAG, Preliminary conversations with <Site 1> staff, Service Eval Observations, National Leaders workshop, Selecting Rooms observation) - PFI buildings – very hard to make changes as all needs to go via landlords (Barrier, Ward isolation solutions meeting observation) - Unprepared IT systems difficult to layer AI (Barrier, Preliminary conversations with <Site 1> and Lister staff) - Connectivity issues within hospitals (Barrier, PAG) - Not great communication among staff- e.g., lack of knowledge of who placed filters or that a study is going on so they cannot switch off filters as happened in previous studies (Barrier, Preliminary conversations with <Site 1> staff, Selecting Rooms observation) - Culture: Difficulty getting people to lead projects that have proven to be beneficial (Barrier, Preliminary conversations with <Site 1> staff) - Aligned with Trusts’ goals of keeping patients and staff safe (Enabler, Preliminary conversations with <Site 1> staff) - Implementation setting - more difficult in inpatients (Enabler/Barrier, Preliminary conversations with <Site 1> staff) - Presence of some mitigation strategies (Enabler, Preliminary conversations with <Site 1> staff) |
|  |  |
| **Characteristics of individuals** | - Responding to the need of reducing infections and protecting staff and patients (Enabler PAG, National Leaders, Preliminary conversations with <Site 1> staff) - Staff wanting to see benefit for them and the organization (Enabler, Preliminary conversations with <Site 1> staff) - HEPA (unclear effectiveness RCTs missing, some filters better than others) (Barrier, Preliminary conversations with <Site 1> staff, National leaders) - Staff supportive with the approval of the clinical lead (Enabler, Preliminary conversations with <Site 1> staff) - Open to anything that helps (Enabler, Preliminary conversations with <Site 1> staff) - More receptive in future pandemics (Enabler, National leaders, Preliminary conversations with <Site 1> staff) - Increased awareness of biological hazards and ventilation since Covid (Enabler, Preliminary conversations with <Site 1> staff) - Lack of preventative approach from staff (Barrier, Preliminary conversations with <Site 1> staff) - Resistance to change (Barrier, Preliminary conversations with <Site 1> staff) |
|  |  |
| **Process** | - Planning maintenance/cleaning plan of mitigation strategies (Barrier, Preliminary conversations with <Site 1> staff) - Engaging national leaders, Board members, Senior members of IPC, local senior champions (Enabler, Preliminary conversations with <Site 1> staff) - Ongoing evaluation and refinement of the AI software (Enabler/Barrier, Preliminary conversations with <Site 1> staff) |
