# Supplementary material for: Anticipated Barriers and Facilitators to the Implementation of an AI Tool Advising on the Placement of Mitigation Strategies to Improve Air Quality in the Hospital Setting: Qualitative Study
Source: JMIR Hum Factors. 2026 Oct 13;13:e93420. doi: 10.2196/93420 (PMC13660969; doi:10.2196/93420)
Supplement: Supplementary file 3 [file humanfactors-v13-e93420-s003.doc]

**Observation sheet**

| Date |  |
| --- | --- |
| Time |  |
| Hospital |  |
| Observation’s location (e.g., Approx percentage of observations made in office, corridor, consulting room, ward...) |  |
| Researcher location in the room (draw location in the space in respect to staff member) |  |
| Others joining observation space (who & consented/not consented?) |  |
| Content of interactions of the staff member observed with any other staff member regarding keeping the hospital air safe from airborne disease transmission following oral consent where possible. |  |
| Tasks carried out (+ which ones on relation to keeping the hospital free from airborne disease transmission) | |
| Further observations | |
