# Supplementary material for: Anticipated Barriers and Facilitators to the Implementation of an AI Tool Advising on the Placement of Mitigation Strategies to Improve Air Quality in the Hospital Setting: Qualitative Study
Source: JMIR Hum Factors. 2026 Oct 13;13:e93420. doi: 10.2196/93420 (PMC13660969; doi:10.2196/93420)
Supplement: Supplementary file 4 [file humanfactors-v13-e93420-s004.doc]

**WP1 - Semi structured interviews with staff**

**Topic guide**

**Role**

- What is your **role** in keeping the hospital free from airborne disease transmission?
- What is the **relevance of ventilation** in your day-to-day work? (e.g., rating 1 –10)
- How long have you been in this post?
- Can you tell me about any **difficulties** you encounter in your day-to-day work to reduce airborne disease transmission within the hospital?
- How could these be addressed?

**Mitigation strategies acceptability**

- What **mitigation strategies**, if any, do you have **currently** in place within the hospital?
- How do you feel about those?
- What are the benefits of having them in place?
- What are the disadvantages?
- What are the reactions among staff and patients to these strategies?
- Are different strategies used in different hospital spaces? How are they different per hospital spaces?
- [If yes] What are the mitigation strategies used within consulting rooms? Which ones are used in outpatient areas?
- [If yes] What are the mitigation strategies used within aerosol generating procedure rooms?
- [If yes] What are the mitigation strategies used within wards?
- Are there any **other mitigation strategies** that you know and would welcome in the hospital? If so, what are those and why would you like to test/use them in the hospital?
- Are there any mitigation strategies that you know of and would **not welcome** in the hospital?
- How would you **facilitate the implementation/installation** of these and similar mitigation strategies within the hospital environment?
- What would help you want to use them or use them more?

**AI tool acceptability**

We are developing an AI tool that guides hospital staff on where to place mitigation strategies (i.e., filters, screens, fans, etc.) within consulting rooms, aerosol generating procedure rooms, and hospital wards. The hospital staff would need to input data into the tool such as the dimensions of the room and where patients and HCPs are positioned. Based on this information the AI tool will share a recommendation on where to position mitigation strategies in the room.

- How does the aim of this AI tool (i.e., help reducing airborne disease transmission) fit with the overall goals of the trust?
- How would the AI tool and the mitigation strategies affect your work? In what ways?
- How would you feel about working in an environment that is relying on AI recommendations to position mitigation strategies?
- What advantages would you expect?
- What would be the disadvantages?
- In what context do you feel the AI tool could be useful? (e.g., a pandemic?)
- Who do you think will find the AI tool more useful?
- How likely is it that they will be willing to put an effort in implementing and adopting the AI tool?
- What, if any, are your concerns about using an AI tool alongside mitigation strategies for staff and patients within the hospital?
- How could these be overcome?
- What would help you want to use the AI tool whilst at work?
- What, if anything, would help you trust the AI tool better?

**Responsibility**

- Who do you think should lead on using the AI tool and placing the mitigation strategies?
- Who should be maintaining the AI tool?
- Who should be cleaning the mitigation strategies?
- If this AI tool was found effective, who would be deciding whether it is going to be implemented within the hospital?
- Who would be funding it?
- What aspects need to be addressed so that it becomes an attractive offer for the hospital/area funding it?

**Feasibility**

- What do you think are the things that could help to roll out this AI tool and the use and re-positioning of mitigation strategies in your hospital?
- What are the barriers to rolling out this AI tool and the use and re-positioning of mitigation strategies in your hospital?
- Are there any ways to overcome these barriers at your hospital?
- How compatible/incompatible do you think is the introduction of the AI tool and the mitigation strategies to the current workload within the teams?
- What training, if any, would staff need to use this AI tool?
- Who should receive this training (e.g., all or only some members of staff)?
- In what format would that training work best?

[Explore relevant aspects observed/discussed via informal conversations]

**End**

- Do you have any questions for us?
- Are you happy for us to contact you again if we have any questions?
- Many thanks for your time and willingness to help us out.
